# Topographic and Morphometric Study of the Foramen Spinosum of the Skull and Its Clinical Correlation

**DOI:** 10.3390/medicina58121740

**Published:** 2022-11-28

**Authors:** Gustavo Tenório Sugano, Carolina Chen Pauris, Yggor Biloria e Silva, Fabrício Egídio Pandini, Raíssa Balabem Said Palermo, Daniela Vieira Buchaim, Rogerio Leone Buchaim, Erivelto Luís Chacon, Cynthia Aparecida de Castro, Bruna Trazzi Pagani, Marcelo Rodrigues da Cunha

**Affiliations:** 1 Department of Morphology and Pathology, Jundiaí Medical School, Jundiaí 13202-550, Brazil; 2 Anatomy Department, Padre Anchieta University Center (UniAnchieta), Jundiaí 13210-795, Brazil; 3 Department of Surgery (Otorhinolaryngology), Jundiaí Medical School, Jundiaí 13202-550, Brazil; 4 Postgraduate Program in Structural and Functional Interactions in Rehabilitation, University of Marilia (UNIMAR), Marília 17525-902, Brazil; 5 Teaching and Research Coordination of the Medical School, University Center of Adamantina (UNIFAI), Adamantina 17800-000, Brazil; 6 Department of Biological Sciences, Bauru School of Dentistry (FOB/USP), University of Sao Paulo, Bauru 17012-901, Brazil; 7 Graduate Program in Anatomy of Domestic and Wild Animals, Faculty of Veterinary Medicine and Animal Science, University of Sao Paulo, Sao Paulo 05508-270, Brazil; 8 Laboratory of Inflammation and Infectious Diseases, Department of Morphology and Pathology, Federal University of Sao Carlos, Sao Carlos 13565-905, Brazil; 9 Dentistry School, University of Marilia (UNIMAR), Marília 17525-902, Brazil

**Keywords:** skull, foramen spinosum, anthropometry, anatomical variations, topography, head and neck, sphenoid bone

## Abstract

*Background and Objectives*: The spinous foramen (FS) of the skull is an opening located in the greater wing of the sphenoid bone at the base of the skull, and it includes the middle meningeal vessels and the meningeal branch of the mandibular trigeminal nerve. The FS is commonly used as an anatomical landmark in neurosurgical procedures and neuroimaging of the middle cranial fossa because of its relationship with other cranial foramina and surrounding vascular and nervous structures. Thus, specific knowledge of its topography and possible anatomical variations is important regarding some surgical interventions and skull imaging. The aim of this study was to provide further details on the morphology of the FS of the skull by evaluating its topographic and morphometric relationships and correlating the findings with clinical practice. *Materials and Methods*: Thirty dried skulls of human skeletons from body donors from the collection of the Laboratory of Anatomical Microdissection at a medical school were used. The metric dimensions and variations of the FS and its relationship with adjacent bone structures were analyzed with an interface digital microscope. *Results*: The results showed the bilateral presence of the FS in all skulls; however, differences were observed in the shape, diameter, and topography in relation to the foramen ovale and the spine of the sphenoid. The FS was present in the greater wing of the sphenoid bone; however, in one skull, it was located in the lateral lamina of the pterygoid process. The FS was smaller than the foramen ovale. A round and oval FS shape was the most common (42.1% and 32.8% of the samples, respectively), followed by drop-shaped (12.5%) and irregular-shaped (12.5%) foramina. *Conclusions*: In conclusion, FS variations among individuals are common and must be considered by surgeons during skull base interventions in order to avoid accidents and postoperative complications.

## 1. Introduction

The foramen spinosum (FS) was described by Jakob Benignus Winslow in the 18th century and was given its name because of its proximity to the spine of the sphenoid bone [1]. It is an opening located in the greater wing of the sphenoid bone as part of the middle cranial fossa [2]. Vascular and nervous structures emerge through this foramen, including the middle meningeal artery (MMA) and the meningeal branch of the mandibular nerve, or the nervous spinous, which originates in the sensory portion of the trigeminal nerve [1,3,4]. The venous component, which is the middle meningeal vein that connects the cavernous sinus with the pterygoid venous plexus, also passes through this foramen [5].

**Figure 1:**
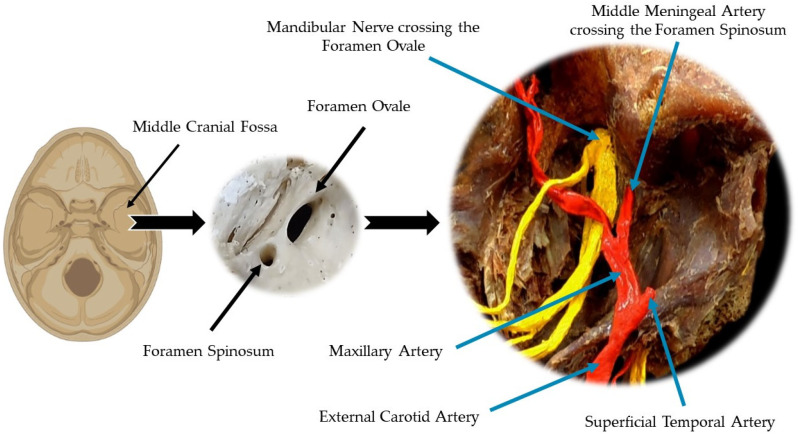
The greater wing of the sphenoid bone is one of the main bone constituents of the middle cranial fossa that, among its various anatomical features, presents the spinous foramen and the foramen ovale. When located close to the temporomandibular joint (TMJ), the external carotid artery gives off its terminal branches, including the superficial temporal artery and the maxillary artery. The maxillary artery can be divided into four portions: mandibular, muscular, maxillary, and pterygopalatine. In the first portion, the mandibular, located in the infratemporal or zygomatic fossa, gives off the middle meningeal artery, which penetrates the skull through the spinous foramen. The mandibular nerve, functionally classified as a mixed nerve, crosses the foramen ovale and distributes its branches in the infratemporal fossa.

The MMA arises from the first portion of the maxillary artery, enters the skull through the FS, travels laterally through a middle fossa bony ridge, and curves over the anterior greater wing of the sphenoid, irrigating the bone and dura mater during its trajectory [6,7,8]. This artery consists of two branches, including an anterior (frontal) branch that passes through the inner surface of the pterion and curves to the apex of the skull and another posterior (parietal) branch that runs along the posterior surface of the skull [9,10]. However, an abnormal morphology of the FS can result in anatomical variations of the MMA which directly interfere with surgical interventions involving the middle cranial fossa [8] (Figure 1).

Vasodilation of the MMA and other intracranial blood vessels has been indicated as a trigger of migraines; therefore, knowledge of their anatomical variations associated with the morphology of the FS is essential. Other clinical situations, such as skull base fractures, epidural hematomas, pterygoid canal nerve surgery, and endovascular interventions of the internal maxillary artery, are also associated with the topography of the MMA and FS [8,11,12] since absence of the FS can alter the development, trajectory, and origin of this artery. Anatomical knowledge of the FS and MMA is also important in cases where there is a possibility of creating a bypass from the MMA to the petrous portion of the internal carotid artery for the treatment of patients with high-flow cervical vascular lesions and infratemporal fossa tumors [13].

Anatomical variations of the FS can be related to incomplete osteogenesis or abnormal development of the MMA [14]. Possible alterations include the formation of two openings because of early branching of the MMA [15]. However, there are also reports of the FS being hypoplastic or absent when the MMA is related to persistence of the stapedial artery, which is a rare congenital vascular anomaly [16,17,18]. This complexity of FS anatomy and its neurovascular content can hinder some surgical procedures involving the middle cranial fossa. Thus, a more detailed understanding of the anatomy of the FS is of great interest in the medical field, especially in neurosurgery, head and neck surgery, and otolaryngology, and it can contribute to the diagnosis of skull base traumas, epidural hematomas, and tumors [19,20,21,22].

The FS is a landmark in middle cranial fossa surgery [1]. However, there is a shortage of data on its normal anatomy and anatomical variations [23], and there is a shortage studies that investigate the association of its morphology with individual characteristics, such as gender, race, ethnicity, and biotype. Surgeons need to be familiarized with these concepts because anatomical variations in the cranial foramina accompanied by possible alterations of the trajectory of associated nervous and vascular structures are common in the population and data are not always readily available. The lack of such knowledge can lead to misinterpretation during clinical evaluation of the middle cranial fossa by computed tomography, which can be confused with abnormalities or diseases. Hence, knowledge of the FS is important in diagnostic medicine [24].

With clinical relevance, the FS is an easily identifiable landmark in middle cranial fossa microsurgery, and knowledge of its anatomical variations is very important for neurosurgeons, radiologists, and anatomists, along with the help of currently available imaging tests. In this way, clinicians can be assisted in diagnosing any aneurysm or other form of vascular injury to the cranial cavity, and this knowledge is useful for neurosurgeons to identify and preserve neurovascular structures when planning approaches to the middle cranial fossa [25].

We realized the need to carry out this study because there are previous studies that describe the variations of FS types [8,14,17,23,24], but there is a lack of data relating these anatomical variations to sex and ethnicity, as well as studies carried out in multiethnic countries, such as Brazil. Thus, this study can be seen as a multiethnic study, complementing previous studies. The aim of the present research was to analyze the variations and dimensions of the FS and correlate the findings with the characteristics of the human skeletons used in this study and with clinical practice.

## 2. Materials and Methods

Thirty human skeleton skulls from body donors obtained from the Anatomical Microdissection Laboratory at the Faculty of Medicine of Jundiaí (FMJ) in Jundiaí, São Paulo, Brazil were used. The Ethics Committee on Research Involving Humans of FMJ approved the study on 1 June 2022 (approval number 58879322.9.0000.5412). Fifteen skulls were from male subjects and the other fifteen were from female subjects. Ages ranged from 22 to 97 years. Seventeen skulls were from white individuals, while ten were from brown individuals, and three were from black individuals.

**Figure 2:**
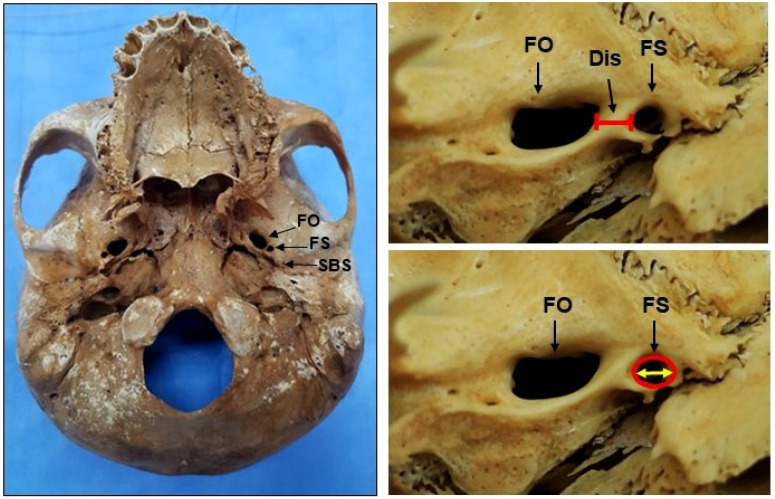
Inferior view of the skull showing the morphometric evaluation (red outline and yellow arrow) of the foramen spinosum (FS) and its distance (Dis) from the foramen ovale (FO) and spine of the sphenoid bone (SBS).

**Figure 3:**
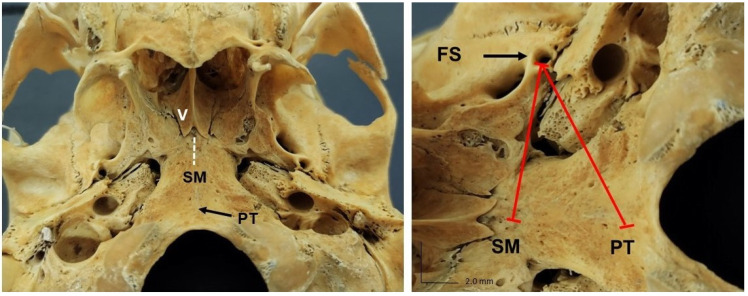
Inferior view of the skull showing the distance from the foramen spinosum (FS) to the midpoint of the skull base (SM) and the pharyngeal tubercle (PT). V: vomer bone.

The skulls were submitted to morphological analysis of the FS and analysis of its dimensions in relation to the foramen ovale (FO), spine of the sphenoid bone, the midpoint of the skull, and the pharyngeal tubercle (Figure 2 and Figure 3). These measurements were obtained using a Dino-Lite^®^ digital microscope equipped with a USB interface (model AM313T, AnMo Electronics Corporation, New Taipei City, Taiwan) at 20× to 200× magnification and a definition of 640 × 480 pixels using the Dino Capture^®^ 2.0 software. This approach obtained accurate metric measurements of the FS of the sphenoid bone in the middle cranial fossa.

The FS was measured at the transverse plane from an inferior view of the skull base as a 2D image using a digital microscope (Dino-Lite^®^). The relevant data included the shape, size, and number of the FS, so there was no need to study the FS in other planes, such as frontal or sagittal.

The measurements obtained were imported to Microsoft Excel^®^ (Microsoft, Redmond, WA, USA) spreadsheets to obtain the average values of the dimensions of the foramen spinosum (FS) on the left and right side and its distance from the foramen ovale (FO), midpoint of the skull base (SM), and pharyngeal tubercle (PT). For the quantitative analysis of the collected data, all tests were performed using GraphPad Prism version 5.00 for Windows (GraphPad Software^®^, San Diego, CA, USA). First, the data obtained were subjected to a normality test (Kolmogorov–Smirnov) and homogeneity of variance (Bartlett’s test). Then, the data were submitted to the paired “*t*” test to compare the results obtained in the measurements of the right vs. left sides. The data were also submitted to the unpaired “*t*” test to compare the results obtained in the measurements of male vs. female skulls (*p* ≤ 0.05).

## 3. Results

This study evaluated the size, symmetry, shape, and other anatomical variations of the FS in 30 skulls obtained from body donor skeletons. The results obtained were compared with data from other studies, and some important differences and similarities were observed. We believe that the present results will provide surgeons and radiologists with a more detailed understanding of the anatomy of the FS and will thus help identify the anatomical landmarks more accurately during surgeries that use a middle cranial fossa approach. The skulls analyzed in this study did not show any type of pathology that would cause bone alterations or deformities.

**Figure 4:**
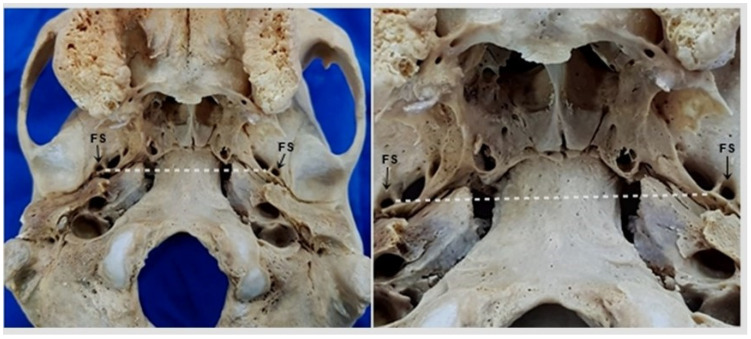
Inferior view of the skull. Note the position and symmetry of the foramen spinosum (FS) between the right and left sides.

Symmetry and bilateral presence of the FS were observed in the studied skulls. The FS was located in the greater wing of the sphenoid in the middle fossa of the skull base, anteromedial to the mandibular fossa of the temporal bone and posterolateral to the FO, with the FS being completely separated from the FO (Figure 4).

**Figure 5:**
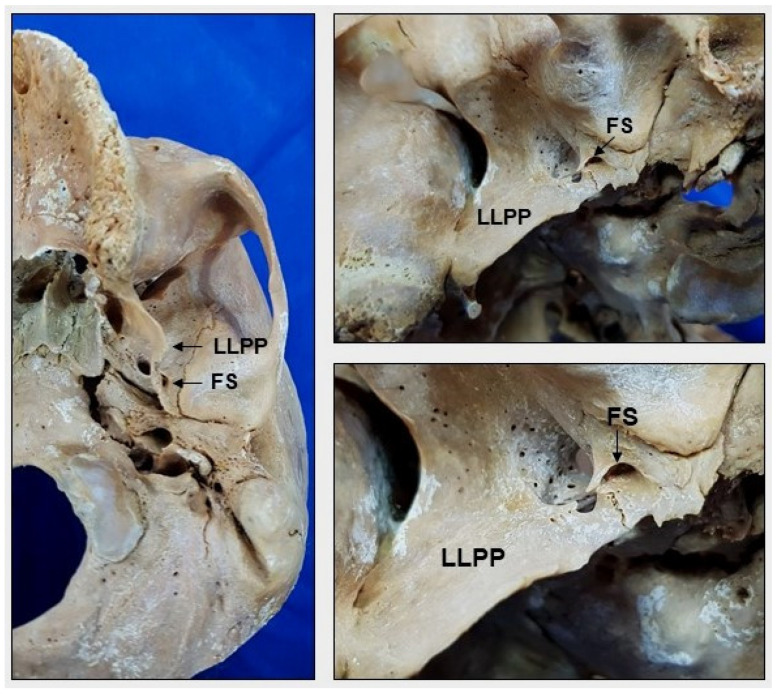
Inferior view of the skull. Note the presence of the foramen spinosum (FS) in relation to the lateral lamina of the pterygoid process (LLPP) of the sphenoid bone.

However, in one skull, the FS was located on both sides of the projection of the lateral lamina of the pterygoid process of the sphenoid bone, which was closer to the transition of the petrotympanic and petrosquamous sutures through the skull base (Figure 5).

**Figure 6:**
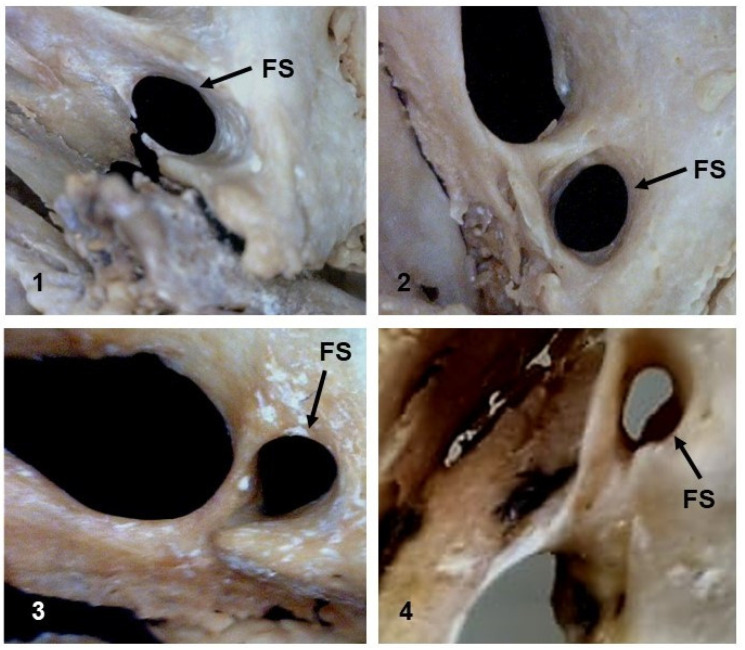
Inferior view of the skull showing the different shapes of the foramen spinosum. Oval (**1**), round (**2**), drop (**3**), and irregular (**4**).

An analysis of the morphology of the FS revealed four different shapes: round, oval, drop, and irregular. The FS was smaller than the FO, and a round and oval shape was the most common (42.1% and 32.8% of the samples, respectively), followed by drop-shaped (12.5%) and irregular-shaped (12.5%) foramina (Figure 6).

**Figure 7:**
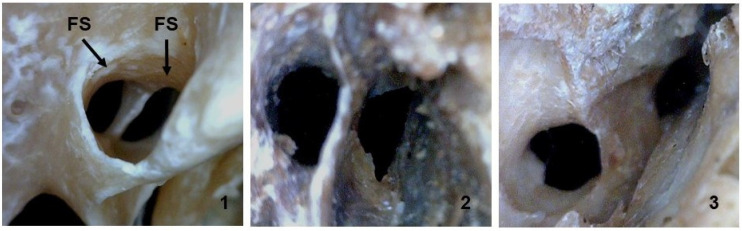
Inferior view of the skull. Note the division (**1**) and duplication in different shapes (**2**,**3**) of the foramen spinosum (FS).

With respect to duplicated or divided FS, this anatomical variation predominated on the left side and was found in three female skulls and one male skull (Figure 7), which corresponded to 13.3% of the samples.

**Figure 8:**
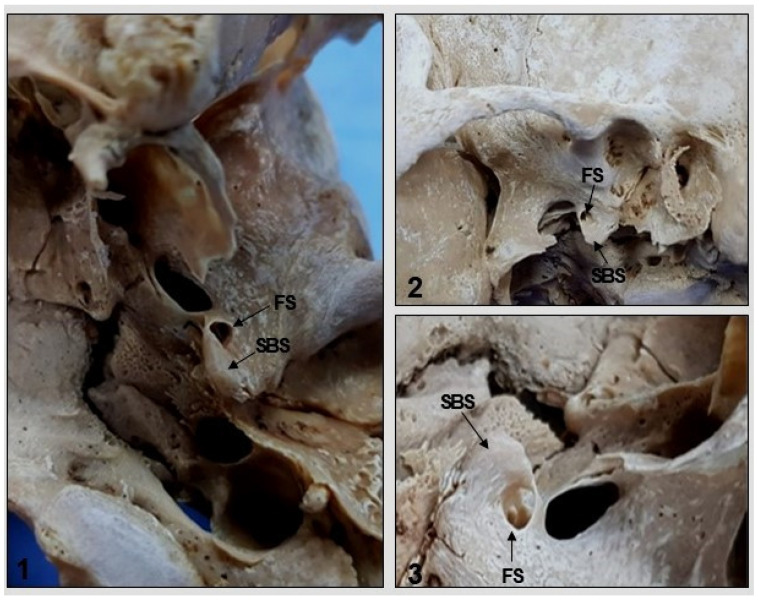
Inferior (**1**) and lateral (**2**,**3**) view of the skull. Note the medial position of the spine of the sphenoid bone (SBS) in relation to the foramen spinosum (FS).

Another anatomical variation observed in two samples was the presence of the sphenoidal spine medial to the FS (Figure 8), while the lateral position was normal and was observed in most skulls. Symmetry of the right and left FS in relation to the median plane of the skull and pharyngeal tubercle was observed in all samples.

An analysis of FS diameter and length and FS–FO distance showed differences in the mean values of the right and left sides, and the differences were more evident in females. In the skulls of female skeletons, the diameter of the FS ranged from 1.68 to 4.70 mm (with a mean of 2.45 mm on the right side and 2.89 mm on the left side). The length of the FS ranged from 5.30 to 14.78 mm (with a mean of 7.72 mm on the right side and 9.09 mm on the left side). The FS–FO distance ranged from 0.36 to 5.24 mm (with a mean of 1.88 mm on the right side and 2.68 mm on the left side). The highest values of all measurements were found on the left side (Table 1 and Table 2).

In male skulls, the diameter of the FS ranged from 1.99 to 2.43 mm (with a mean of 2.69 mm on the right side and 2.62 mm on the left side). The length of the FS ranged from 6.26 to 10.79 mm (with a mean of 8.48 on the right side and 8.26 mm on the left side). In contrast to female skulls, higher values of the two measurements were observed on the right side. Regarding FS–FO distance, the values ranged from 0.5 to 5.76 mm (with a mean of 2.87 on the right side and 2.97 on the left side). As observed in female skulls, the measures were higher on the left side. There was no significant difference in the distance of the FS to the midpoint of the skull base or pharyngeal tubercle between the right and left sides in either gender (Table 1 and Table 2).

In the statistically analyzed results, there was no significant difference between the right and left sides or difference between the male and female skulls (*p* ≤ 0.05).

## 4. Discussion

The incidence of anatomical variations in cranial foramina in terms of diameter, symmetry, presence or absence, and unilateral or bilateral presentation is still debatable. However, this information is essential because of the advancements in diagnostic imaging methods used to assess pathological conditions that affect these foramina and their neurovascular content [23]. Detailed knowledge of variations in cranial foramina is also necessary to preserve neurovascular structures during middle cranial fossa surgeries [1] performed in cases of pathologies that cause obstruction of the cranial nerve passage and potentially severe clinical consequences. Despite the evidence of the importance of cranial foramina, a comprehensive anatomical review of these structures is still necessary, which is a fact that justifies the need for further studies on their morphology [26].

The FS is an important structure located in the base of the skull that is generally posterolateral to the FO and anteromedial to the spine of the sphenoid bone [27]. The morphological development of the FS starts when infants are eight months old and completes by seven years of age [28]. Important vascular structures that irrigate the dura mater arise from the FS [29]. Thus, in-depth studies of the FS can assist with new medical techniques used to treat middle cranial fossa traumas and tumors [30].

In the present study, the FS was located in the greater wing of the sphenoid bone in all skulls. However, there are reports of the presence of this foramen in the squamous part of the temporal bone or in the sphenosquamosal suture [14,31]. The presence of the FS in the greater wing of the sphenoid observed in the skulls studied aligns with the literature. However, in one skull, the FS was located bilaterally in the projection of the lateral lamina of the pterygoid process of the sphenoid bone, which is a variation that is not identified in the scientific publications consulted for this study. This finding is important for the interpretation of radiological images, such as computed tomography scans performed in head traumas.

There is an important relationship between the FS and the spine of the greater wing of the sphenoid, which is usually located posterolateral to this foramen [32]; however, a medial location has also been described [1]. Studying 40 adult skulls, Sophia and Kalpana (2015) found the FS bilaterally in an anteromedial position to the sphenoidal spine in 25% of the samples, with 30% on the right side and 33.75% on the left side [24]. In addition, this foramen was located laterally to the sphenoidal spine in 26.25% of the skulls and medial to the sphenoidal spine in 3.75%. The authors reinforced the importance of this information for neurosurgeons in order to avoid damage to the meningeal artery and its accompanying vein when approaching the base of the skull.

In the present study, the sphenoidal spine was found to be located both posteromedial and posterolateral to the FS. In two skulls, the sphenoidal spine was located posteromedial to the foramen which corresponded to 6.66% of the samples and is a percentage higher than the number reported in a similar study involving other populations [32]. Such variations can affect the course of the middle meningeal vessels and can cause damage to the auriculotemporal or chorda tympani nerve [25].

The FS was present bilaterally in the 30 skulls used in this study. However, there is evidence of its unilateral absence. For example, Berge and Bergman (2001) described the unilateral absence of the foramen in one (1%) of 100 skulls studied [23]. Nikolova et al. (2012) observed the unilateral absence of the FS in one (0.70%) male skull and in one (0.72%) female skull on the right side and in three (2.13%) female skulls on the left side [14]. In one (0.72%) female skull, a small FS was located atypically on the right side [14]. Nikolova et al. (2012) reported no cases of bilateral absence of the FS, while Lindblom (1936) observed that this foramen was small or completely absent in 0.4% of the samples, especially in cases where the MMA originated from the ophthalmic artery [33].

The FS is found in up to 99% of cases. However, its unilateral [23] or bilateral [34] absence and its duplication [35] has been reported in the literature, which is even more evident depending on the population studied. In the study by Worku and Clarke (2021), the FS was absent in two of the 64 studied skulls (3.12%) and in 3/128 sides (2.34%) and was duplicated in one of the 64 skulls examined (1.56%) and in 1/128 sides (0.78%) [32]. Sophia and Kalpana (2015) studied 40 dry adult human skulls, and the FS was absent in two (2.5%) skulls on the left side and duplicated in three (3.75%) [24].

We found no unilateral or bilateral absence of the FS, which corresponds with Teul et al. (2002) [36], but the foramen was duplicated in 13.3% of the samples. Lindblom (1936) also reported the presence of a double FS [33]. Ginsberg et al. observed the absence of the FS in 3.2% (*n* = 123) of patients [35]. Nikolova et al. (2012) reported the complete absence of the FS in five cases or a small size in combination with an atypical position in one case, which was associated with variations in the origin and trajectory of the MMA [14]. Naqshi et al. (2017) observed confluence of the FO and FS on the left side in one of 20 skulls analyzed, duplication of the FS on the right side separated by a thin bone plate in one case, and its absence on the right side in one skull [37].

Duplication of the FS is the result of the development of a thin or thick bone plate that divides the foramen into two compartments in an event that increases the possibility of the early division of the MMA into anterior and posterior branches in the infratemporal fossa [38,39]. In the present sample, the FS was duplicated in four (13.3%) skulls, including two skulls from white female patients, one skull from a brown female patient, and one skull from a white male patient, all on the left side.

According to Tewari et al. (2018), the FS can exhibit different shapes, with a round shape being the most common (55.56%), followed by oval-shaped (33.33%), drop-shaped (7.14%), and irregular-shaped (3.96%) foramina [38]. Worku and Clarke (2021) observed a round shape of the FS in 50% of cases (64/128), an oval shape in 32.81% of cases (42/128), a drop shape in 10.94% of cases (14/128), and an irregular shape in 6.25% of cases (8/128) [32]. In the study by Sophia and Kalpana (2015), who analyzed 40 dry adult human skulls, a round-shaped FS was the most common (52.5%), followed by oval-shaped (30%), irregular-shaped (12.5%), and drop-shaped foramina (2.5%) [24]. Taken together, these findings indicate that an oval or round shape of the FS is usually the most common [40].

Four types of FS shapes were observed in the present study of adult skulls, including an oval shape in 42.1%, a round shape in 32.8%, a drop shape in 12.5%, and an irregular shape in 12.5%. Our data thus differ from the literature as Yanagi (1987) reported a predominance of round-shaped FS [41].

The present study revealed differences in the FS measurements, with higher values on the left side in female skulls and on the right side in male skulls. Berge and Bergman (2001) demonstrated that 14/92 (15%) skulls studied had asymmetric FS (size difference > 0.5 mm), with a similar incidence on the right and left sides [23]. However, the incidence of asymmetry greater than 1.5 and 1.0 mm was only 1% and 2%, respectively [23]. In contrast, the 30 skulls evaluated in the present study exhibited symmetry of the FS with the median plane and pharyngeal tubercle on both sides.

Other studies have reported differences in FS diameters, including diameters ranging from 2.39 to 1.96 mm [23], a diameter of 2.63 mm in adults [41], diameters of 3.72 and 3.37 mm on the right and left sides, respectively [32], and an average diameter of 2 mm [33]. Studying 80 skulls, Somesh et al. (2015) found a mean length, mean width, and mean area on the right side of the FS of 3.42 mm, 2.68 mm, and 7.35 mm^2^, respectively, and of 3.33 mm, 2.67 mm, and 7.11 mm^2^ on the left side, respectively; however, there was no significant difference between the sides, despite the higher values on the right side [42]. In a study by Rai et al. (2012), the mean length of the FS in male skulls was 3.31 mm on the left side and 3.73 mm on the right side, while in female skulls, the mean length was 3.20 and 3.81 mm on the left and right sides, respectively. However, there was no significant difference between sides or sexes [43]. Lang et al. (1984) reported a mean length of the FS of 2.25 mm in newborns and of 2.56 mm in adults. Its width extended from 1.05 to about 2.1 mm in adults [40].

In the study by Sophia and Kalpana (2015) of 40 adult human skulls, the mean length of the FS was 3.96 mm on the right side and 4.25 mm on the left side, while the mean width was 2.21 mm on the right side and 2.18 mm on the left side [24]. Reymond et al. (2005) reported that the mean area of the foramina measured in the skull, including the FS, foramen rotundum, and foramen of Vesalius, but excluding the FO, was not considerable, suggesting that these foramina play a minor role in the dynamics of blood circulation in the venous system of the head [44].

This present study correlated the anatomical and metric variations of the FS with skull side (right and left) and gender (male and female). However, correlation of the data with race was not possible because among the 30 skulls studied, 17 were from white individuals, 10 were from brown individuals, and three were from black individuals, and the races were therefore not proportionally distributed among the skulls. However, this variable should be investigated in future studies since there are reports that the incidence of certain variations is associated with population type, origin, and/or gender [45]. Anatomical differences can be explained by general factors of anatomical variation, such as age, sex, race, and biotype (including longeline, breviline, and midline) [46].

In a case report, Ellwanger and Campos (2013) observed an abnormal shape of the FS in a Caucasian anatomical specimen skull of an approximately 35-year-old individual which was probably due to an abnormality caused by a variation in the trajectory of the MMA [8]. Adachi (1928) observed the absence of the FS in 0.4% (*n* = 800) of Japanese skulls and in 1.4% (*n* = 800) of Russian skulls [47]. Kwathai et al. (2012) studied 103 skulls of Thai origin and observed three FS shapes, including round (49.5%), oval (39.8%), and irregular (10.7%) [48].

Mandavi and Mishra (2009) evaluated 312 skulls from the Japanese population, and the FS was absent bilaterally in one skull and duplicated in eight [49]. Osunwoke et al. (2010) found no skull with an absent FS among 87 skulls obtained from the southern Nigerian population [50]. Somesh et al. (2015) observed a predominance of round-shaped FS among 82 dry adult human skulls of unknown sex of South Indian origin [42], which aligned with another study of South Indian skulls conducted by Kulkarni and Nikade [51]. In addition, these authors also performed a detailed description of the FS in terms of its morphological variations, size, distance from the midline, and other information based on the ethnic origin of the skulls in order provide neurosurgeons, neuroradiologists, and neurologists with data for medical procedures that involve the middle cranial fossa.

Variations in the shape of the FS and its absence are important for vascularization of the encephalic dura mater since the MMA can have an anomalous origin and emerge through the FO, originate from the persistent stapedial artery that arises from the intrapetrous internal carotid artery, or arise from the ophthalmic artery following the lateral portion of the superior orbital fissure [52,53]. According to Manjunath (2001), in addition to its usual origin from the first portion of the maxillary artery, the MMA can also arise from the third portion of the maxillary artery, the stapedial artery, the ophthalmic artery, the cavernous segment of the internal carotid artery, and even the basilar artery [54]. In Manjunath’s study involving 524 sides of dry skulls and 20 dissected anatomical specimens, the MMA originated from the orbit in six cases, and the FS were rudimentary or absent [54]. It is therefore important to know the variations of the FS and MMA in order to understand the embryological bases and their surgical importance. In a case report, Cvetko and Bosnjak (2014) also described a bilateral ophthalmic origin of the MMA and unilateral absence of the FS. According to the authors, identification of the anomalous origin of the MMA is important for planning surgical and endovascular interventions in the middle cranial fossa and orbit [55].

We also investigated the relationship between the FS and FO. This information is important for certain surgeries, such as percutaneous trigeminal rhizotomy and percutaneous biopsy of cavernous sinus tumors, because their procedures can cause damage to the neurovascular structures of the FS due to its proximity to the FO [25,39]. The FS is usually located 3 mm posterolateral to the FO [26]. Worku and Clarke (2021) observed confluence of the FO and FS in one of the 64 skulls examined (1.56%) and in 2/128 sides (1.56%) [32]. Regarding the FS–FO distances obtained in the present study, differences were observed between women and men, with greater mean distances in the latter. Furthermore, the FS were completely separated from the FO in this study. However, studies have reported incomplete separation between these two foramina, with unilateral in 2/100 (2%) and bilateral in 1/100 (1%) of the skulls studied [23].

In view of the above data, it is clear that knowledge of the complex anatomy of the FS and of surrounding bone and neurovascular structures directly influences clinical, radiological, and surgical interventions that explore the skull base. The FS is thus an important landmark in middle cranial fossa surgeries since surgical approaches are technically more difficult without this knowledge. Furthermore, detailed knowledge of the FS for radiologists is essential for the interpretation of abnormal foramina on radiological images [24,56].

The number of skulls that were analyzed and the use of the collection of a single anatomy laboratory can be considered as limitations of this study. With a greater and more diversified collection, the results regarding the relationship and proportion of the anatomical variations of the FS and different races could be strengthened.

## 5. Conclusions

In conclusion, the FS exhibits important anatomical variations in terms of its shape, diameter, and topography in relation to adjacent bone structures. These data are important for better identification and preservation of neurovascular structures during surgical procedures involving the middle cranial fossa of the skull, and they contribute to the clinical anatomical knowledge of physicians, dentists, and anatomists.

## Figures and Tables

**Table 1:** Dimensions of the foramen spinosum (FS) on the right side and its distance from the foramen ovale (FO), midpoint of the skull base (SM), and pharyngeal tubercle (PT).

Female	Minimum	Maximum	Mean
Diameter	1.68 mm	3.23 mm	2.45 mm
Length	5.30 mm	10.15 mm	7.72 mm
FS–FO distance	0.36 mm	3.21 mm	1.88 mm
FS–SM distance	8.8 mm	10.97 mm	9.93 mm
FS–PT distance	9.52 mm	12.25 mm	10.79 mm
Male	Minimum	Maximum	Mean
Diameter	2.27 mm	3.43 mm	2.69 mm
Length	7.14 mm	10.79 mm	8.48 mm
FS–FO distance	0.5 mm	4.32 mm	2.87 mm
FS–SM distance	8.66 mm	10.78 mm	9.99 mm
FS–PT distance	9.35 mm	11.59 mm	10.78 mm

**Table 2:** Dimensions of the foramen spinosum (FS) on the left side and its distance from the foramen ovale (FO), midpoint of the skull base (SM), and pharyngeal tubercle (PT).

Female	Minimum	Maximum	Mean
Diameter	2.10 mm	4.70 mm	2.89 mm
Length	6.60 mm	14.78 mm	9.09 mm
FS–FO distance	0.49 mm	5.24 mm	2.68 mm
FS–SM distance	8.80 mm	10.34 mm	9.68 mm
FS–PT distance	9.13 mm	11.38 mm	10.0 mm
Male	Minimum	Maximum	Mean
Diameter	1.99 mm	3.43 mm	2.62 mm
Length	6.26 mm	10.77 mm	8.26 mm
FS–FO distance	0.79 mm	5.76 mm	2.97 mm
FS–SM distance	8.16 mm	10.78 mm	9.53 mm
FS–PT distance	8.37 mm	11.76 mm	10.06 mm

## Data Availability

Not applicable.
